# Comparative performance of the EuroQol EQ-5D-5L and the CDC healthy days measures in assessing population health

**DOI:** 10.1186/s41687-022-00474-7

**Published:** 2022-06-13

**Authors:** Maryna Derkach, Fatima Al Sayah, Arto Ohinmaa, Lawrence W. Svenson, Jeffrey A. Johnson

**Affiliations:** 1 Alberta PROMs and EQ-5D Research and Support Unit (APERSU), School of Public Health, University of Alberta, 2-040 Li Ka Shing Centre for Health Research Innovation, Edmonton, AB T6G 2E1 Canada; 2 Alberta Health, Station Main, PO Box 1360, Edmonton, AB T5J 2N3 Canada

## Abstract

**Objectives:**

To examine the comparative performance of EuroQol EQ-5D-5L and Center for Disease Control Healthy Days measures in assessing population health.

**Methods:**

Using data from 2014 Alberta Community Health Survey, a cross-sectional population-based survey (N = 7559), conducted in Alberta, Canada, we examined construct validity of the measures as indicators of population health. Differences in EQ-5D-5L index score, visual analogue scale (EQ-VAS), and CDC unhealthy days index across socio-demographic subgroups were tested by Mann–Whitney and Kruskal–Wallis tests using known-groups approach.

**Results:**

EQ-5D-5L and CDC Healthy Days provided comparable assessments of population health in this sample. Both measures discriminated between subgroups defined by self-perceived health status, level of education, and material deprivation. The discriminative ability of CDC Healthy Days was limited in capturing variability in health among age groups compared to the EQ-5D-5L. Among participants who reported 0 unhealthy days, the proportion of those with level 3 problems in pain/discomfort varied from 1.1% for participants aged 18–24 to 19.2% for those over 75 years.

**Conclusions:**

EQ-5D-5L demonstrated better construct validity than CDC Healthy Days in assessing health in a population-based sample of adults.

## Introduction

Population-based surveillance of physical and mental health may be based on different measures, which vary by purpose and concept. Traditional measures of population health, such as mortality rates, diseases incidence and prevalence address the burden of the disease and disability through the objective assessment of health. Though informative, these measures fall short in capturing the patients’ perspective on their own health, whether or not they have any conditions or treatments. Further, those traditional indicators do not reflect the impact of patient’s health status on the quality of life and well-being. Currently many countries are moving towards patient-centered systems of structuring, monitoring, and delivering health care, which is generating the interest in using patient-reported outcome measures (PROMs). There is growing evidence that PROMs are valid indicators of population health [1–3], useful for measuring the impact of the chronic disease, and the outcomes of health care interventions [4–6] and other purposes.

Increasing demand for PROMs have led to the development of a number of generic and disease-specific measurement tools. Among the well-recognized and broadly used measures are the EuroQol EQ-5D and the United States Center for Disease Control and Prevention (CDC) Healthy Days. The former is preference-based health related quality of life measure (HRQoL) which is widely used in research, public health policy and clinical setting across the world. The latter is brief, not preference-based health assessment measure that is extensively used in the U.S.^1^ and other countries.

The underlying constructs and methodologies of the measures are different, which may raise concerns about complete interchangeability of the measures. However, both tools are generic and multidimensional, captures physical and mental aspects of health and designed to measure self-reported health related quality of life of groups of patients or general population. The motivation of the research was to shed light on the comparative performance of EQ-5D-5L and the CDC Healthy Days in regards of the accuracy of the public health assessment and comparative advantages and limitations of their usage in public health surveillance, policy and research.

There is extensive literature comparing the EQ-5D with other measures, such as the SF-6D [7–11]; Quality of Well-Being (QWB) and Health Utilities Index (HUI) [11, 12]; and the WHO Quality of life questionnaire 5 items (WHOQOL-5) [13]. Many studies found that EQ-5D-5L and SF-6D has similar evidence of the discriminative validity in population of patients^2^ [7, 8, 10], and showed that SF-6D appeared to be more responsive to larger improvements in symptoms [7] or was more efficient in detecting differences among subgroups with differing health status [8]. Other studies documented that EQ-5D-5L performed better for some disease conditions (skin conditions, some types of cancer) and worse for others like hearing impairments [9]; and displayed significant ceiling effect [12]. On the other hand, few studies have compared the performance of the CDC Healthy Days and EQ. Lubetkin and Jia examined the comparability of responses of these two measures in the United States general population [14]. They used data from two independently drawn U.S. population samples and concluded that sociodemographic and some clinical variables had similar effects on mean values of indices and scores of the CDC Healthy days and EQ-5D-3L. Our study aimed to expand this analysis by comparing the performance of these measures in assessing population health applied to one population sample and using the new 5 level version of the instrument, EQ-5D-5L.

## Methods

Data from the Alberta Community Health Survey (June 2014 cycle) were used in this analysis. The survey is a cross-sectional study that collects information related to health status, health related quality of life, risk factors and determinants of health of adult population of Alberta^3^ Sampling design of the study included sampling quotas and randomized selection of dial numbers. Sampling quotas were drawn from the Alberta Health Service (AHS) zones^4^ to obtain high quality, geographically representative estimates [18]. The sampling quotas were representative of the age and gender composition of each AHS zone and were based on 2013 estimates of the population in each AHS zone provided by the Alberta Ministry of Health. The survey was conducted using computer-assisted telephone interview (CATI) with random digit dialing to ensure that each household had an equal chance of being contacted to participate in the survey. The target population included adults, who is at least 18 years old and reside in Alberta. Out of a total of 187,672 people meeting the eligibility criteria and pre-selected for interview 26,515 (14.1%) were defined as indeterminate, 127 450 (67.9%) were disqualified, and 25 666 (13.6%) refused to participate in the study. Overall, 7,559 Albertans aged 18 and older completed the survey with a response rate of 23.0%, which is lover than for some other community health surveys [19] but comparable with the other surveys conducted by telephone interview [20, 21].^5^

### Measures

The EQ-5D is a generic preference-based measure for describing and valuing health-related quality of life [22]. The data elements of the EQ-5D-5L include the health profile, EQ VAS score, and index score. EQ-5D health profile is a description of person’s current self-reported health state using 5 dimensions: mobility, self-care, usual activities, pain/discomfort, and anxiety/depression. In the EQ-5D-5L each dimension has 5 levels of problems: 1 “no”, 2 “mild, 3 “moderate”, 4 “severe”, 5 “extreme,” describing 3125 distinct health states [23]. Visual analog scale (VAS) score ranges between 0 (labeled ‘worst imaginable health state’) and 100 (‘best imaginable health state’) and records person’s overall self-rated health. EQ-5D index score (also referred as utility score) is the value assigned to each EQ-5D health state to reflect how good or bad the health state is according to the preferences of the general population. The index scores were obtained using the EQ-5D-5L population-based value set for Canada (range − 0.148 to 0.949) developed in Canadian 5-L valuation study [24].

The CDC Healthy Days is a brief health related quality of life measure designed to assess person’s perceived sense of well being through four-item questionnaire. The first question relates to individuals’ self-perceived health as “a state of complete physical, mental and social well-being and not merely the absence of disease or infirmity” [25]. It asks participants to rate their health on a scale from poor to excellent. The second and third questions assess the recent physical and mental health asking for how many days during the past 30 days a respondent’s physical (second question) or mental (third question) health was not good. Question 4 pertains to individual’s incapacity due to physical or mental problems and asks for how many days during the past 30 days a respondent’s poor physical or mental health kept her from doing her usual activities such as self-care, work or recreation. Additionally, the CDC introduced the summary measure, Unhealthy Days Index (UDI) aiming to capture the concept of the total number of days with impairment. It is an estimate of the overall number of days during the previous 30 days when the respondent’s physical or mental health was not good. The index is calculated by summing the responses to the physically unhealthy and mentally unhealthy days (questions two and three). If the sum is greater than 30, a maximum score of 30 is assigned. The index is based on assumption that logical overlap of reported physically, and mentally unhealthy days is minimum [2].

### Statistical analysis

Descriptive statistics (mean, standard deviation (SD), minimum and maximum values, or median and interquartile ranges (IQR)) were computed for all variables as appropriate. The associations between the EQ-5D-5L and the CDC Healthy Days scores were examined using Spearman correlation. The performance of both measures was compared regarding their construct validity based on the criteria for review of generic HR-QoL instruments developed by the Scientific Advisory Committee of the Medical Outcomes Trust [26, 27]; and based on studies that outlined the importance of the link between measurement properties and the purpose of the health-related quality of life measures. According to Guyatt et. Al., (1992) validity and reproducibility are considered as a most rigorous approach to compare the validity of discriminative measures [28]. Construct validity of both measures was assessed using the known-groups approach, with subgroups defined by a self-perceived health status, age, education, and levels of material deprivation. The latter was defined by Canadian deprivation index (CDI) [29]. The greater value of index relates to the higher level of deprivation, starting with level 1 (“the least materially deprived”) to level 5 (“the most materially deprived”). Due to the skewed and homoscedastic distribution of the EQ-5D-5L and Healthy Days measure scores, between-group comparisons were performed using Mann–Whitney and Kruskal–Wallis tests as appropriate.

### Construct validity

In applying the known groups approach, we compared mean and median values of UDI, EQ-5D-5L index score and EQ-VAS among the subgroups of the population based on age, self-reported health status, level of educational attainment, and the CDI category. The discriminative properties of the measures were assessed using the following hypotheses: 1) median values of the EQ-5D-5L index score and EQ-VAS would be higher for participants who rated their health as “very good or excellent” compared to those who rated their health as “good, fair or poor”, while median values of UDI would be lower for the former group compared to the latter; 2) participants with a higher level of education would have higher median of EQ-5D-5L index and VAS score and lower those for the UDI; 3) health status of the individuals is different across age groups [30, 31]. In addition, folowing the literature on the relation between health and level of personal welfare [32], we hypothesized that scores would be lower for the UDI and higher for EQ-5D-5L index and EQ-VAS for the groups with lower values of Canadian deprivation index (less materially deprived).

Construct validity of the CDC Healthy Days was further examined by comparing the proportion of participants who experienced 0, 1–9, 10–19, 20–29, 30 unhealthy days between the subsamples defined according to their self-rated health. Our hypothesis was that the proportion of people who experienced many unhealthy days (30 or 20–29) would be higher among those who rated their health as “poor” or “fair” compared to those whose health was stated as “excellent” or “very good”. Correspondingly, proportion of people with 0 unhealthy days will be lower among the participants with poorer health status.

Next, using the EQ-5D-5L dimensions scores, we analyzed the health profiles of participants with zero days of impairment (0 unhealthy days) and a full month of impairment (30 unhealthy days) for the groups of population stratified by age. The purpose was to understand the extent to what the health condition of participants who report the minimum value of unhealthy days corresponds to full health; and contrarily how does the maximum value of unhealthy days relate to the state of extreme impairment based on the EQ-5D-5L.

### Ceiling and floor effects

As mentioned in the literature, ceiling effect is common phenomenon observed in both health profile and preference-based measures when score distribution tends to be skewed to the left [33]. Ceiling effect makes it difficult to detect changes in the score among subjects among the top end of the scale and, therefore is important criteria to compare health related quality of life measures. Ceiling and floor effects for EQ-5D-5L index score and the CDC Healthy Days were defined as the proportion of participants with the best and worst theoretical scores, respectively. For the EQ-5D-5L, the best score associated with level 1 “no problem” on all dimensions (health state 11111); and the worst score with level 5 “extreme problem” on all dimensions (health state 55,555). For the index and items of Healthy Days measure, the worst score corresponds to 30 days when either physical or mental health was not good, or when usual activity was limited due to health-related problems. Contrarily, the best score was represented by zero number of days of impairment or limited functioning.

All analyses were conducted using STATA (Stata Corp. 2015. Stata Statistical Software: Release 15. College Station, TX: Stata Corp LP).

## Results

Of 7559 participants who completed the survey, 61.0% were female, 90.7% had high school education or more (Table 1). Distribution of the study sample across the age groups, was comparable with those of adult population of Alberta and Canada, with slight underrepresentation of young adults, 18–34 years old, (20.9% in the study sample vs. 33.8% in Alberta and 23.1% in Canada); and over-representation of 75 + age group (8.32% vs. 6.2% in Alberta and 6.8% in Canada) [15]. 72.7% of participants were in level 1or 2 of CDI, and 2.2% were in level 5.

**Table 1:** General characteristics of participants of the study

	N (%)
**Age groups (N = 7477)**	
18–24	471 (6.3)
25–34	1,092 (14.6)
35–44	1,210 (16.2)
45–54	1,344 (18.0)
55–64	1,565 (20.9)
65–74	1,173 (15.7)
75 +	622 (8.3)
**Gender (N = 7559)**	
Females	4,612 (61.0)
Males	2,947 (39.0)
**Education (N = 7495)**	
Below high school	699 (9.3)
High school	4,698 (62.7)
University	2,098 (28.0)
**Self-perceived health status**^**a**^**(N = 6558)**	
Excellent	1,450 (22.1)
Very good	2,538 (38.7)
Good	1,691 (25.8)
Fair	589 (9.0)
Poor	290 (4.4)
**Canadian deprivation index (N = 6324)**	
The least materially deprived	1425 (22.5)
Well off, but not the most well off	3174 (50.2)
Less well off, sort of deprived	1206 (19.1)
Quite materially deprived	378 (5.9)
The most materially deprived	141 (2.2)

^a^This variable represents the first item of Healthy Days measure asking the respondents to rate their health on a five categories scale

### EQ-5D-5L and the CDC Healthy Days measures

Respondents in this sample reported 416 different EQ-5D-5L health states (13% of possible 3125 states). Mean (SD) of EQ-5D-5L index score was 0.849 (0.142), ranging from − 0.0911 to 0.949, with a distribution skewed to the right (perfect health). Median (IQR) value was 0.904 (0.121). The ceiling effect was 29.6%, and the floor effect was 0. Mean and median values of EQ-VAS were 79.5 (17.7) and 85 (15.0) respectively; with a distribution positively skewed towards best imaginable health. Ceiling effects for EQ-5D-5L Mobility, Self-Care, Usual Activities, Pain/Discomfort and Anxiety/ Depression dimensions were 75.8%, 93.7%, 74.6%, 40.6%, 64.4%. Floor effects were 0.5%, 0.1%, 1.0%, 1.0%, 0.9%.

Overall, participants’ perception of their health ranged from good to excellent (86.6%), with only 13.4% reporting fair to poor health (Table 1). Mean (SD) of the Healthy Days measure domains were 4.7 (8.6) for physically unhealthy days, 3.8 (7.3) for mentally unhealthy days, 2.9 (6.9) for days with activity limitation, and 7.4 (10.5) for the UDI. Median values (IQR) of UDI and Healthy Days measure domains were equal to 2.0 (10.0) for UDI, 0 (5.0) for physically unhealthy days; 0 (4.0) for mentally unhealthy days; 0 (2.0) for days with activity limitation. Ceiling and floor effects for the UDI were 34.5% and 11.9%, respectively. For unhealthy days physically, unhealthy days mentally, and days with activity limitation ceiling effects were 51.4%, 53.4%, 70.5%, and floor effects were 7.1%, 4.0%, and 3.6%.

We found moderate correlations between the UDI and EQ-5D-5L index score (r = -0.56) and between the UDI and EQ-VAS (r = -0.48). In general, the correlations between Healthy Days items and EQ-5D-5L dimensions and index were found to be from negligible to moderate, ranging from 0.15 to 0.63 in absolute value. The highest values of Spearman coefficient were observed for self-rated health and EQ-VAS (r = -0.67), and the lowest for self-care dimension and unhealthy days mentally (r = 0.15).

### Construct validity of EQ-5D-5L and the CDC Healthy Days

As hypothesized, participants who perceived their health as “very good or excellent” had fewer unhealthy days, higher EQ-5D-5L index score and higher EQ-VAS than the other subgroup (Table 2). One way analysis variance test by ranks (Kruskal–Wallis test) performed for Unhealthy days index, EQ-5D-5L index score and EQ-VAS allowed to reject the null hypothesis for each of the dependent variables (*p* value < 0.01), implying that in each case the age groups subpopulation did not came from the same distribution and both Unhealthy days index and EQ-5D-5L index score and EQ-VAS scale were statistically different across age groups. Median values of the EQ-5D-5L index score and EQ-VAS were lower for older age groups of the population. Median values of Unhealthy days index were decreasing between youngest and oldest age groups but was at the same level for ‘25–34 ‘,’35–44’,’45–54’ age groups (median 3 days) and for’55–64’, ‘65–74’, ‘75 + ’ groups (median 2 days).

**Table 2:** The CDC Healthy Days and EQ-5D-5L measures by sociodemographic descriptions

	Unhealthy days index		EQ-5D index score		EQ-VAS	
	Mean(SD)	Median(IQR)	Mean(SD)	Median(IQR)	Mean(SD)	Median(IQR)
**Age group**						
18–24	8.1 (9.3)	4 (11)	0.883 (0.1)	0.911 (0.082)	83.3 (14.0)	85 (10)
25–34	7.0 (9.2)	3 (10)	0.887 (0.103)	0.911 (0.082)	82.8 (15.4)	85 (13)
35–44	6.7 (9.1)	3 (8)	0.881 (0.107)	0.905 (0.082)	82.1 (15.1)	85 (15)
45–54	8.1 (10.8)	3 (11)	0.843 (0.154)	0.905 (0.121)	78.6 (19.2)	82 (15)
55–64	8.0 (10.9)	2 (12)	0.825 (0.167)	0.87 (0.122)	78.3 (18.5)	80 (20)
65–74	7.3 (10.5)	2 (10)	0.821 (0.151)	0.865 (0.122)	77.0 (19.4)	80 (20)
75 +	6.5 (9.7)	2 (8)	0.799 (0.161)	0.846 (0.147)	74.7 (18.7)	80 (25)
*p*-value	0.001		< 0.001		< 0.001	
**Education**						
Below high school	9.7 (11.5)	5 (17)	0.788 (0.189)	0.860 (0.142)	72.9 (21.2)	80 (30)
High school	7.9 (10.4)	3 (11)	0.843 (0.145)	0.904 (0.126)	79.1 (17.8)	80 (15)
Certificate/ University	5.6 (8.6)	2 (6)	0.882 (0.105)	0.904 (0.082)	83.0 (15.2)	85 (11)
*p-value*	< 0.001		< 0.001		< 0.001	
**Self-Rated Health**						
Very Good/Excellent	3.9 (6.7)	1 (5)	0.901 (0.066)	0.913 (0.082)	87.2 (11.2)	90 (20)
Good/Fair/Poor	13.3 (11.9)	10 (28)	0.767 (0.184)	0.827 (0.167)	67.4 (19.2)	73 (15)
*p-value*	< 0.001		< 0.001		< 0.001	
**Canadian Deprivation Index**						
1 The least materially deprived	5.4 (8.4)	2 (6)	0.883 (0.102)	0.905 (0.082)	83.1 (14.8)	85 (10)
2	6.8 (10.4)	2(10)	0.859 (0.122)	0.905 (0.119)	80.7 (16.4)	85 (15)
3	8.8 (10.8)	4 (15)	0.827 (0.159)	0.867 (0.110)	77.0 (19.2)	80 (20)
4	13.3 (12.0)	10 (28)	0.763 (0.218)	0.860 (0.214)	70.8 (23.3)	75 (35)
5 The most materially deprived	16.9 (12.3)	19 (25)	0.707 (0.247)	0.803 (0.307)	64.8 (24.5)	70 (30)
*p-value*	< 0.001		< 0.001		< 0.0010	
Sample mean(SD) median (IQR)	7.4 (10.5)	2 (10)	0.849 (0.142)	0.905 (0.121)	79.5 (17.7)	85 (25)

**Fig. 1:**
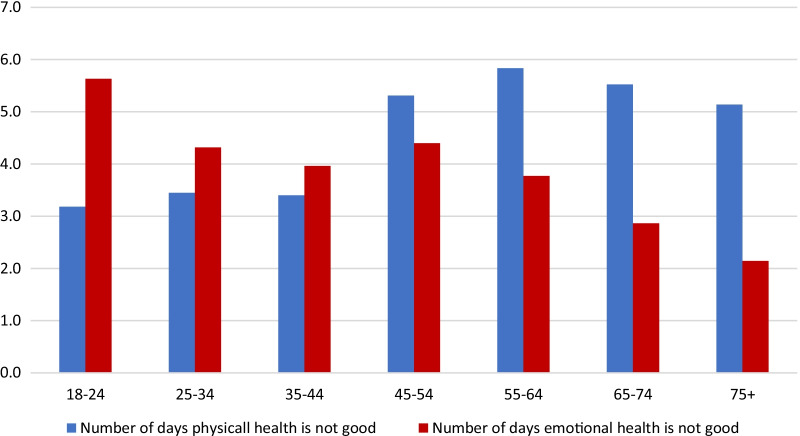
Average number of days when physical/emotional health is not good by age groups

**Fig. 2:**
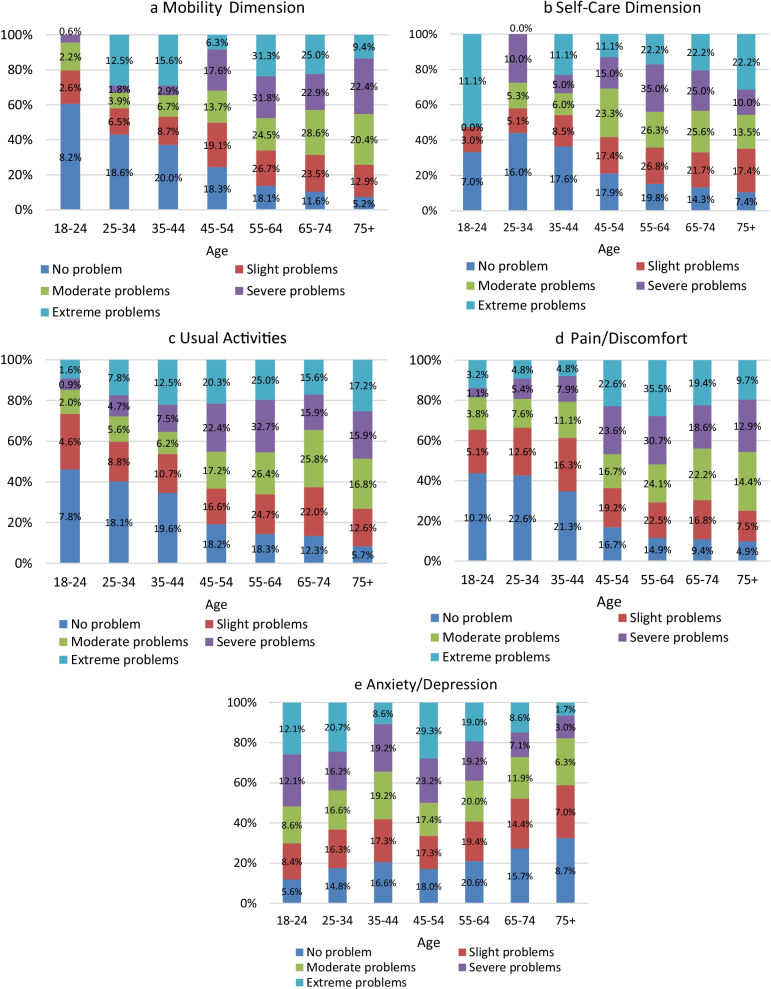
EQ-5D-5L dimension scores by age groups

The direction of change of unhealthy days physically and unhealthy days mentally items of Healthy Days measure across age groups showed that health status of the population was worsening with older age in physical domain, while improving in the mental/emotional health domain (Fig. 1). Analogous pattern was identified for EQ-5D-5L dimensions. The proportion of population with level 1 (‘No problem’) decreased with age in the Mobility, Self-Care and Pain/discomfort domains of EQ-5D-5L and increased in the Anxiety/Depression dimension (Fig. 2).

Based on the levels of education and of the CDI, the mean and median values of EQ-5D-5L index score and EQ-VAS were higher for subgroups with higher education and lower deprivation index, while the mean and median of the UDI were lower for these subgroups. The effect size for the difference between “below high school” and “certificate/university” level of education was equal to 0.40, 0.60 and 0.54 for UDI, EQ-5D-5L index score and EQ-VAS respectively. The effect size for the difference between the subgroups with the lowest and the highest value of CDI was − 1.09 for UDI, 0.903 for the EQ-5D-5L index score, and 0.904 for the EQ-VAS.

The proportion of the population who reported 0, 1–9, 10–19, 20–29, 30 unhealthy days varied between subgroups of the population who rate their health from “excellent” to “poor.” 56.0% of the population who rated their health as “excellent” reported 0 unhealthy days, 35.6% had 1–9 unhealthy days, 5.1%, 1.5%, and 1.8% indicated 10–19, 20–29 and 30 unhealthy days correspondingly (Table 3). For those who perceived their health status as “very good” the proportion of 0 healthy days was 36.7%, while the proportion of 1–9, 10–19, 20–29 and 30 unhealthy days was 47.1%, 9.7%, 2.6%, and 3.8%, accordingly. Among the group who rated their overall health as “poor,” only 3.0% reported 0 unhealthy days, although 67.1% recorded 30 unhealthy days.

**Table 3:** General characteristics of participants by number of unhealthy days

	**Number of unhealthy days**					*p*-value
	30	20–29	10–19	1–9	0	
Overall (N = 7,311)	873(11.9)	321(4.4)	786(10.8)	2812(38.5)	2519 (34.5)	
**Age groups**						< 0.001
18–24	36 (7.8)	38 (8.2)	76 (16.5)	206 (44.7)	105 (22.8)	
25–34	90 (8.4)	54 (5.1)	139 (13.0)	478 (44.6)	311 (29.0)	
35–44	97 (8.0)	54 (4.6)	129 (10.9)	537 (45.3)	369 (31.1)	
45–54	198 (15.1)	50 (3.8)	132 (10.1)	489 (37.3)	442 (33.7)	
55–64	235 (15.4)	62 (4.1)	154 (10.1)	495 (32.5)	577 (37.9)	
65–74	152 (13.5)	39 (3.5)	102 (9.0)	388 (34.4)	448 (39.6)	
75 +	58 (10.5)	22 (3.9)	51 (9.2)	185 (33.4)	238(42.9)	
**Gender**						< 0.001
Females	558 (12.6)	218 (4.9)	544 (12.3)	1,766 (39.7)	1,359 (30.6)	
Males	315 (11.0)	103 (3.6)	242 (8.4)	1,046 (36.5)	1,160 (40.5)	
**Education**						< 0.001
Below high school	125 (19.1)	36 (5.5)	77 (11.8)	198 (30.3)	217 (33.2)	
High school	588 (12.9)	221 (4.9)	513 (11.3)	1,693(37.2)	1,531 (33.7)	
University	150 (7.3)	62 (3.0)	190 (9.24)	906(44.1)	748 (36.4)	
**Self-perceived health status**						< 0.001
Excellent	26 (1.8)	22 (1.5)	73 (5.1)	509 (35.6)	798 (55.9)	
Very good	96 (3.8)	66 (2.6)	243 (9.7)	1,178 (47.1)	917 (36.7)	
Good	237 (14.5)	109 (6.7)	273 (16.7)	674 (41.3)	339 (20.8)	
Fair	230 (42.2)	65 (11.9)	87 (12.6)	117 (16.0)	46 (8.4)	
Poor	180 (67.1)	29 (10.8)	16 (6.0)	35 (13.1)	8 (3.0)	
**Canadian deprivation index**						
The least materially deprived	95 (6.8)	46 (3.3)	121 (8.7)	640 (45.7)	499 (35.7)	< 0.001
Well off, but not the most well off	316 (10.2)	124 (4.0)	342 (11.0)	1220 (39.4)	1096 (35.4)	
Less well off, sort of deprived	174 (15.0)	68 (5.9)	128 (11.0)	451 (39.0)	338 (29.2)	
Quite materially deprived	96 (26.5)	28 (7.7)	60 (16.6)	110 (30.4)	68 (18.8)	
The most materially deprived	50 (37.1)	17 (12.6)	16 (11.9)	32 (23.7)	20 (14.8)	

### EQ-5D-5L dimensions for 0 and 30 unhealthy days population subgroups

**Fig. 3:**
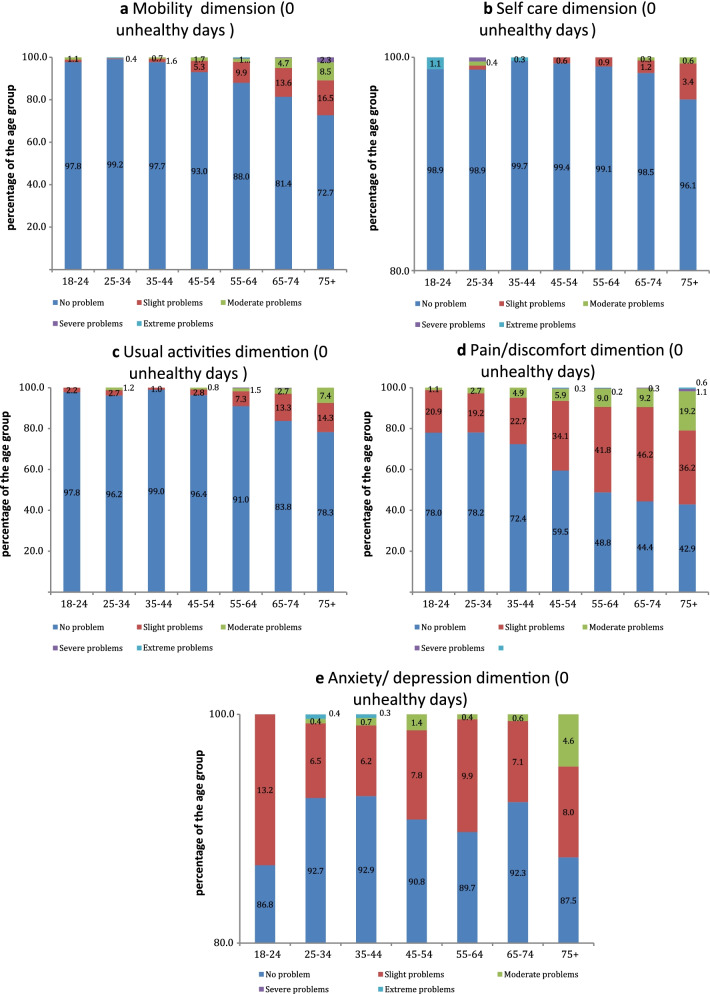
EQ-5D-5L dimensions scores for the population subgroup of 0 unhealthy days by age groups

Among the participants who reported 0 unhealthy days the proportion of respondents who reported levels 1–5 of health problems in each of the EQ-5D-5L dimensions varied significantly between age groups, especially in mobility and pain/discomfort dimensions (Fig. 3). For pain/discomfort, 22.0% of participants aged 18–24-year-old reported Level 2 problem and higher, while for the 45–54, 55–64, 65–74 and 75 + age groups, this proportion amounted to 40.5%, 51.2%, 55.6%, and 57.1% respectively. The proportion of respondents who indicated moderate problems related to pain/discomfort was 1.1% for the age group 18–24 and 19.2% for the 75 + -year subgroup. In the mobility dimension, the percentage of participants who reported higher than level 1 problems were 1.1%, 0.4%, and 1.6% for 18–24, 25–34, 35–44 years old, respectively. For older population it reached 7.0%, 12.0%, 18.6%, and 27.3% for 45–54, 55–64, 65–74 and 75 + age groups (Fig. 3a). The proportion of the population who reported higher than level 1 problems both in mobility and pain discomfort dimensions was higher for the participants with 0 unhealthy days physically or 0 unhealthy days mentally.

**Fig. 4:**
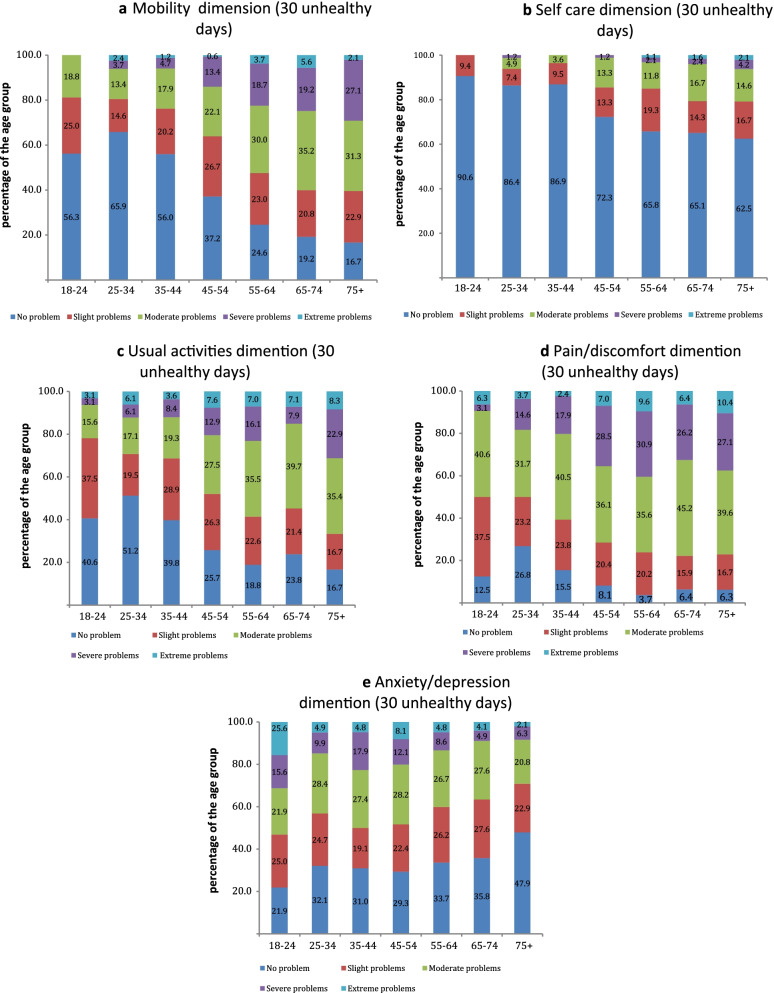
EQ-5D-5L dimensions scores for population subgroup of 30 unhealthy days, by age groups

EQ-5D-5L health profiles of the participants who reported 30 unhealthy days showed that in pain/discomfort, the percentage of the participants who reported no problems varied from 6.3% (65–74 and 75 + age groups) to 26.8% (25–34-year-old) (Fig. 4). The proportion of participants with moderate problems was 31.7% and higher for all age groups. In the mobility dimension, the percentage of participants who reported level 1 problem ranged from 56.0% to 65.9% for the population younger than 45 and was significantly lower for other age groups. In general, the proportion of participants who reported 11111 health state was 2.2% (19 people out of 873 participants with 30 unhealthy days).

### Healthy Days measure items responses for population in ‘11111’ EQ-5D-5L health state

Among the respondents who reported perfect health state (‘11111’ health profile of EQ-5D-5L) the percentage of population with 0 days when health was not well was equal 58.2%, 73.7%, 73.7%, 89.5% for Unhealthy days index, unhealthy days physically, unhealthy days mentally and number of days when activity was limited, respectively (Table 4). The percentage of population who reported 21–29 or 30 days when health was not good, or activities were limited due to health problem was less than 1 percent for all items of the CDC Unhealthy days and Unhealthy days index.

**Table 4:** Unhealthy Days Index and Items of Healthy Days Measure for the Population Subgroup of ‘11111’ Health State of EQ-5D-5L

	N (count)	%
**Unhealthy Days Index**		
0	1075	58.2%
1–10	681	36.9%
11–20	56	3.0%
21–29	12	0.6%
30	19	1.0%
N	1847	100
**Number of days when physical health is not well**		
0	1367	73.7%
1–10	444	23.9%
11–20	28	1.5%
21–29	5	0.3%
30	11	0.6%
N	1855	100
**Number of days when mental health is not well**		
0	1366	73.7%
1–10	457	24.6%
11–20	17	0.9%
21–29	9	0.5%
A	5	0.3%
N	1854	100
**Number of days when activity was limited**		
0	1666	89.5%
1–10	180	9.7%
11–20	12	0.6%
21–29	1	0.1%
30	3	0.2%
N	1862	100

## Discussion

In comparing the performance of the EQ-5D-5L and the CDC Healthy Days measures in assessing population health, we found that there was moderately strong association between summary indexes of measures, EQ-5D-5L index score and Unhealthy days index. Both measures can effectively differentiate between population subgroups by self-rated health status, education, and level of material deprivation. Additionally, our analysis showed that the measures performed differently in the overall health assessment of different age subgroups; with the EQ-5D-5L demonstrating slightly better construct validity compared with the CDC Healthy Days.

Our findings provide further evidence of good construct validity of the EQ-5D-5L measure, as reported in the previous studies [8, 34]. Median values of EQ-5D-5L index score and EQ-VAS were higher for subgroups who reported fewer unhealthy days. Our results are also consistent with studies that documented good construct validity of the CDC Healthy Days [35–37]. The individuals who rated their health as “excellent” less frequently reported 30 or 20–29 unhealthy days and more frequently 0 unhealthy days compared to those who rated their health as “poor” or “fair.” Analysing the pattern of change of EQ-5D-5L index score, EQ-VAS and the UDI across the population subgroups we found that participants with a higher level of education, lower level of material deprivation were characterized with better health which agrees with the literature [14, 36].

Our results indicated that the measures performed differently in the overall health assessment of different age subgroups. Both mean and median values of EQ-5D-5L index score and EQ-VAS were lower for older age groups, supporting the findings from studies [10, 14, 38, 39]. The direction of change of mean and median values of the CDC unhealthy days index was not consistent across age groups. The median values of UDI were overall higher for the younger age groups and lower for the older groups, implying that health status of population improves with age. Mean values of UDI changed non-linearly among age groups.

Neither of the measures aligned with the literature showing worsening of the physical health component and improvement of mental health with older age [14, 40]. However, this pattern was well identifiable in EQ-5D-5L dimensions, and Healthy Days measure items, supporting previous findings [14, 40].

Our results indicate that EQ-5D-5L provided slightly more accurate assessment of population overall health compared to the CDC Healthy Days. The analysis of EQ-5D-5L health profiles among the age subgroups of the population who reported 0 or 30 days of impairment showed that interpretation of 0 unhealthy days scores varied significantly among age groups. When applied to the younger population, the CDC Healthy Days and EQ-5D-5L showed similar results and characterized the population health status as close to full health. However, for older groups, there was a significant portion of the population who experienced level 2 and higher health problems in pain/discomfort or mobility dimensions.

There were several limitations to our study to note. First, the generalizability of the results was restricted to the identical population sample. The data were collected through telephone interviews with high non-response rate, typical for this mode of the survey. It is likely that some subgroups of the population, like people in poor health condition, the institutionalized population were not adequately represented. Second, the known-group analysis was performed based on socio demographic characteristics of the population, while variables reflecting participant’s objective physical and mental health were not available.

It would be beneficial direction of further research to compare performance of EQ-5D-5L and the CDC Healthy Days in clinical setting using data on the sample of patients with disease conditions manifested with older age. It would be also interesting to extend analysis to comparison of the performance of the two measures across gender groups. Third, our analysis was based on cross-sectional data collected in one cycle of the community health survey, limiting possibility to compare reliability and responsiveness of the measures. Analysis of longitudinal data set could open new venues for the research, allowing to perform more extensive analysis of core measurement properties of HRQoL instruments, and also, to analyse changes in population health status in time.

## Conclusion

The results of our study indicate that both EQ-5D-5L and the CDC Healthy Days provide a relatively comparable assessment of overall population health and represent efficient tools for the surveillance of health-related quality of life. Both measures can be equally useful for the purposes of identification of health disparities among demographic and socioeconomics subgroups and monitoring of general population health. However, our findings showed that EQ-5D-5L had slightly better construct validity than the CDC Healthy Days and would provide more detailed picture of population health status related to age. For this reason, EQ-5D-5L could be considered as more favorable tool in the areas of health policy and research related to surveillance of health-related quality of life of older age groups of population. That may include tracking of health status, patient related outcomes of treatments, estimation of the burden of the disease conditions and utilization of health services and others. Also, EQ-5D-5L would allow to obtain richer results of population health status when combined with geriatric studies, or administered in special setting, such as health programs and services dedicated for care and support of older age people.

## Data Availability

The data that support the findings of this study are openly available in the Open Government Portal (Alberta) at https://open.alberta.ca/opendata/alberta-community-health-survey-achs-telephone-conducted.

## Footnotes

1 Since 1993 the CDC Healthy Days has been included in The Behavioral Risk Factor Surveillance System (BRFSS), a continuous nationwide survey of non-institutionalized the U.S. adult population. In addition, the measure has been included in the National Health and Nutrition Examination Survey, Medicare Health Outcome Survey.

2 Sayah.F. et al. (2017) based their research on data on population of adults with type 2 diabetes in Alberta, Canada [7]; Chen J et. al (2014) studied patients with COPD in China [8].

3 Alberta is a province in Western Canada with the estimated population 4,054,280, median age 36, and total life expectancy 81.1 years at the time of the study [15–17]. In 2014, 51 percent of Albertian population were males, 49 percent were females. The number of people aged 65 and older represented about 11% of the population; Alberta’s working age population (those aged 15 to 64 years) represented about 70% of the total population in 2014 [16].

4 Alberta province includes South, Calgary, Central, Edmonton, and North health zones.

5 The response rate was calculated as the ratio of the number of completed interviews to the number of eligible reporting interviews minus the number of indeterminate responses and minus of disqualified responses.
